# Capacities and needs of health care facilities for schistosomiasis diagnosis and management in elimination settings

**DOI:** 10.1186/s13071-024-06311-8

**Published:** 2024-06-17

**Authors:** Naomi C. Ndum, Lydia Trippler, Ulfat A. Mohammed, Anisa S. Ali, Jan Hattendorf, Jürg Utzinger, Said M. Ali, Stefanie Knopp

**Affiliations:** 1 Swiss Tropical and Public Health Institute, Allschwil, Switzerland; 2 University of Basel, Basel, Switzerland; 3 Public Health Laboratory–Ivo de Carneri, Pemba, United Republic of Tanzania

**Keywords:** Attitude, Control, Elimination, Health facility, Knowledge, Management, Practices, *Schistosoma haematobium*, Signs and symptoms, Tanzania

## Abstract

**Background:**

Schistosomiasis is a debilitating neglected tropical disease endemic in sub-Saharan Africa. The role of health facilities in the prevention, diagnosis, control, and elimination of schistosomiasis is poorly documented. In a setting targeted for schistosomiasis elimination in Zanzibar, we assessed the prevalence of *Schistosoma haematobium* among patients seeking care in a health facility and investigated schistosomiasis-related knowledge of staff, and health facilities’ capacities and needs for schistosomiasis diagnosis and management.

**Methods:**

We conducted a health facility-based mixed-method study on Pemba Island from June to August 2023. Patients aged ≥ 4 years seeking care in four health facilities were screened for *S. haematobium* infection using urine filtration and reagent strips. Those patients aged ≥ 10 years were additionally interviewed about signs and symptoms. Staff from 23 health facilities responded to a questionnaire assessing knowledge and practices. Ten staff participated in a focus group discussion (FGD) about capacities and needs for schistosomiasis diagnosis and management.

**Results:**

The prevalence of *S. haematobium* infection in patients attending the health facilities, as determined by the presence of eggs in urine, was 1.1% (8/712). Microhaematuria was detected in 13.3% (95/712) of the patients using reagent strips. Among patients responding to the questionnaire, pelvic pain, pain during sex, and painful urination were reported by 38.0% (237/623), 6.3% (39/623), and 3.2% (20/623), respectively. Among the health facility staff, 90.0% (44/49) and 87.8% (43/49) identified blood in urine and pelvic pain, respectively, as symptoms of urogenital schistosomiasis, 81.6% (40/49) and 93.9% (46/49) reported collecting a urine sample and pursuing a reagent strip test, respectively, for diagnosis, and 87.8% (43/49) administered praziquantel for treatment. The most reoccurring themes in the FGD were the need for more staff training about schistosomiasis, requests for diagnostic equipment, and the need to improve community response to schistosomiasis services in health facilities.

**Conclusions:**

The prevalence of *S. haematobium* infection in patients seeking care in health facilities in Pemba is very low and similar to what has been reported from recent community-based cross-sectional surveys. The health facility staff had good schistosomiasis-related knowledge and practices. However, to integrate schistosomiasis patient management more durably into routine health facility activities, scalable screening pathways need to be identified and capacities need to be improved by regular staff training, and an unbroken supply of accurate point-of-care diagnostics and praziquantel for the treatment of cases.

**Graphical abstract:**

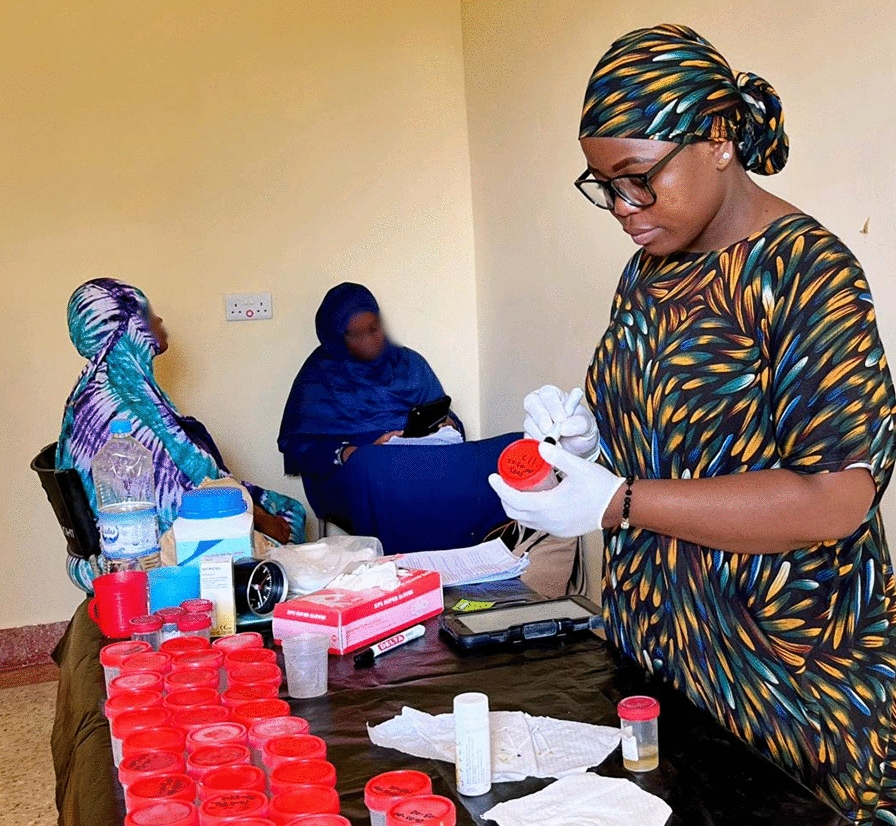

**Supplementary Information:**

The online version contains supplementary material available at 10.1186/s13071-024-06311-8.

## Background

Schistosomiasis is a debilitating neglected tropical disease (NTD) caused by infection with parasitic blood flukes of the genus *Schistosoma* [1]. Schistosomiasis is transmitted in 78 countries and territories worldwide, but most of the burden is concentrated in sub-Saharan Africa [2, 3]. The typical clinical presentation of urogenital schistosomiasis caused by *S. haematobium* is blood in urine [4]. Accompanying signs and symptoms may include painful urination and genital lesions, which may progress to fibrosis and calcification of the bladder and lower ureters, unilateral or bilateral hydroureters, and hydronephrosis [4]. If left untreated, long-term consequences of urogenital schistosomiasis may result in poor growth in children, bladder cancer, infertility, female genital schistosomiasis, and prostrate male genital schistosomiasis [1, 5].

Over the past 2 decades, interventions to control the morbidity due to schistosomiasis have been scaled up; hence, the prevalence and intensity of *Schistosoma* infections have substantially decreased [6, 7]. Several areas are now approaching elimination [8, 9]. Preventive chemotherapy with praziquantel, implemented by national schistosomiasis control programmes, has been the mainstay of schistosomiasis morbidity control since the new millennium [10, 11]. Along with social and economic development, preventive chemotherapy is the key driver for the observed reduction in prevalence [6]. Moreover, environmental management, including water engineering and focal snail control, behaviour change measures, and water, sanitation, and hygiene (WASH) interventions are recommended by the World Health Organization (WHO) for interrupting disease transmission [12]. These interventions are implemented mostly in areas that aim to achieve elimination [13–16].

Studies have pointed out that health facilities could play a role in the control and elimination of schistosomiasis. The studies identified a high prevalence of *Schistosoma* infections and related symptoms among patients attending health facilities [17, 18]. Research also indicated the direct role of health facilities in the management of schistosomiasis in terms of case identification, diagnosis, and treatment [18, 19]. In the new guidelines for the control and elimination of schistosomiasis, published in 2022, WHO recommends that “…health facilities provide access to treatment with praziquantel to control morbidity due to schistosomiasis in all individuals regardless of age, including infected pregnant excluding the first trimester, lactating women and preschool-aged children < 2 years” [12]. In addition, health facilities may contribute to the assignment of targeted interventions for the control and elimination of schistosomiasis and play a role in outbreak investigation and passive surveillance, especially in elimination settings [20, 21]. In an elimination setting, the majority of the population is uninfected and the few infected individuals mainly carry light-intensity infections [22, 23]. In such settings, health facilities can support the WHO targets for the elimination of NTDs by screening, testing, treatment, and reporting cases [2, 24].

Unguja and Pemba, the Zanzibar islands of the United Republic of Tanzania, are considered an elimination setting for urogenital schistosomiasis [2, 25]. *Schistosoma haematobium* is the only endemic schistosome species and, thanks to almost a century of research and control efforts, the overall prevalence is nowadays very low [8, 22, 25]. In the northern part of Pemba, the “SchistoBreak” project has been implemented since 2020, which aims to assess the impact of targeted interventions for schistosomiasis elimination [26]. As part of the SchistoBreak project, a cross-sectional survey conducted in 2021 revealed a very low prevalence of *S. haematobium* among school-aged children (1.2%) and community members (0.8%) [27]. Across the project area, health facilities are involved in the passive surveillance of schistosomiasis [26].

The aim of the current study, readily embedded in the SchistoBreak project, was to determine the potential role of health facility involvement in programmatic elimination efforts. Hence, we assessed the prevalence of *S. haematobium* infections among patients aged ≥ 4 years seeking care at health facilities, determined the schistosomiasis-related knowledge of health facility staff, and examined the capacity and needs of health facilities for schistosomiasis diagnosis, treatment, and management.

## Methods

### Study area

**Fig. 1:**
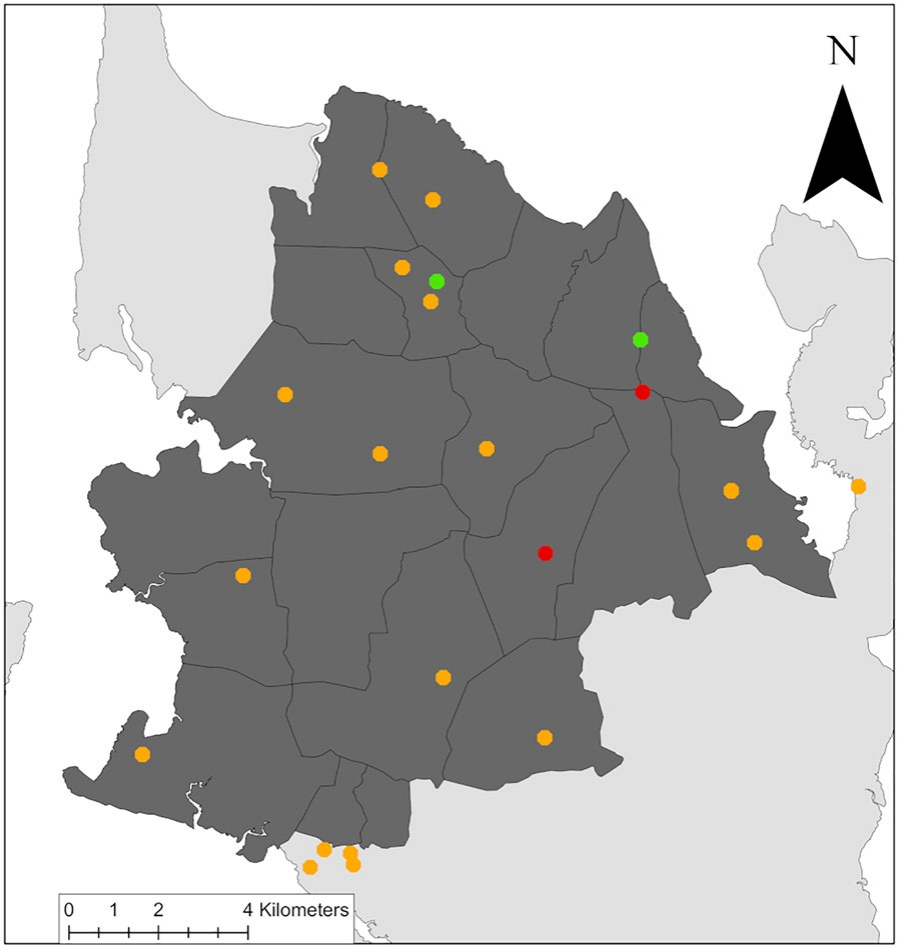
Map of 22 health facilities in the northern part of Pemba involved in our study. The geolocation of one health facility is missing. Light grey: North of Pemba Island; dim grey: 20 small administrative areas (shehias) in the SchistoBreak project area; red/orange/green: 22 health facilities where staff participated in the questionnaire survey; red and green: 2 hotspot and 2 low-prevalence health facilities, respectively, where patients were screened for microhaematuria and *Schistosoma haematobium* infections. The image base map (United Republic of Tanzania—Subnational administrative boundaries) was downloaded from the United Nations (UN) Office for the Coordination of Humanitarian Affairs (OCHA) services (https://data.humdata.org/dataset/tanzania-administrative-boundaries-level-1-to-3-regions-districts-and-wards-with-2012-population). The data source of the image base map is Tanzania National Bureau of Statistics/UN OCHA ROSA. The data of the image base map are published under the following license: Creative Commons Attribution for Intergovernmental Organisations (CC BY-IGO; https://creativecommons.org/licenses/by/3.0/igo/legal code). We received written permission to use and adapt the data from OCHA. Additional shape files for the map (shehia boundaries) were provided by the Zanzibar Commission for Lands to the Zanzibar Neglected Diseases Programme

This study was carried out on the island of Pemba, located in the Zanzibar archipelago of the United Republic of Tanzania, approximately 30 km off the coast from the East African mainland in the Indian Ocean [28, 29]. According to the 2022 national census, Pemba has a population of 543,441 inhabitants [29]. Our study was embedded in the SchistoBreak project, which has been carried out in 20 small administrative areas (shehias) in the northern part of Pemba from 2020 to 2024 [26]. In line with the threshold provided in the study protocol [26], in 2022, the SchistoBreak project area consisted of four *S. haematobium* hotspots (i.e. areas with an *S. haematobium* prevalence of ≥ 3% prevalence in school children or with an *S. haematobium* prevalence of ≥ 2% prevalence in community members aged ≥ 4 years) and 16 low-prevalence areas. In the SchistoBreak project area, there are 23 health facilities [21 primary health care units (PHCUs) and two district hospitals] (Fig. 1). The PHCUs in Tanzania are first-line health facilities focusing on basic health care, maternal-child care, immunization programmes, outreach and health education services, and water and sanitation efforts [30]. The district hospitals serve as second-line referral facilities and provide specialised health care services [31].

### Study design and procedures

This health facility-based mixed-method study was conducted from June to August 2023. The study consisted of two parts. In the first part, a health facility-based survey was conducted in four health facilities aiming to assess the microhaematuria and *S. haematobium* prevalence in all attending patients aged ≥ 4 years. Additionally, signs and symptoms related to urogenital schistosomiasis were determined in all patients aged ≥ 10 years.

The four health facilities were selected based on their location in hotspot or low-prevalence areas (two PHCUs each), patients’ attendance numbers in 2022/2023, and the willingness of health staff to collaborate. Each health facility was visited by the study team for 8 days. On the days of the study team’s visits between 08:00 and 14:00 h, all patients aged ≥ 4 years who had signed a written informed consent form (parental guardians signed for individuals aged < 18 years) received an empty plastic container (100 ml) labelled with a personal identifier code and were invited to submit their own urine sample. Promptly after submission, the urine samples were examined for microhaematuria using Hemastix reagent strip tests (Siemens Healthcare; Zurich, Switzerland) at the point of care. Reagent strips were graded as negative, non-haemolysed trace, haemolysed trace, small, moderate, and high according to the manufacturer’s colour chart. The results of the microhaematuria grading were entered directly at the point of care into tablet computers (Samsung Galaxy Tab A, Samsung Electronics; Seoul, South Korea) using Open Data Kit (ODK) software (www.opendatakit.org). The urine samples were kept in a cool box and transferred to the Public Health Laboratory-Ivo de Carneri the same day. Urine samples were examined for *S. haematobium* infection using a filtration method by experienced laboratory technicians [26]. In brief, 10 ml of urine was pressed through a 13-mm filter (Sefar; Kent, UK) using a plastic syringe with a Swinnex filter holder (Merck; Darmstadt, Germany). The filter was placed on a glass slide and examined under a microscope for the presence of *S. haematobium* eggs. Eggs were counted, recorded on paper forms in the laboratory, and entered twice into a Microsoft Excel spreadsheet (Microsoft 2013 and Microsoft 2016; Redmond, Washington, USA).

All patients aged ≥ 10 years were invited to participate in a questionnaire interview. Data on socio-demographic characteristics and self-reported signs and symptoms that may be related to urogenital schistosomiasis were collected. In the questionnaire (Supplementary file 1), there were a total of 20 questions on signs and symptoms of urogenital schistosomiasis. Among them, four questions were directed to female participants only: (i) Do you often have irregular menstruation? (ii) Do you experience vaginal bleeding? (iii) Are you pregnant? (iv) Are you currently breastfeeding? The latter two questions were restricted to females aged > 15 years. One question—do you experience pain during sex?—was only asked if a patient was > 12 years old, an age when sexual activity may start [32, 33]. Two members of the local research team conducted the questionnaire interviews in the local language (i.e. Kiswahili). Answers were entered directly into a tablet computer using the ODK software.

The second part of the study was a qualitative approach that aimed to assess schistosomiasis-related knowledge and practices of health facility staff. Within the SchistoBreak project, staff from all 23 health facilities in the study area received biannual lectures about urogenital schistosomiasis, updates about the SchistoBreak project, trainings about reagent strip tests for microhaematuria assessment as a proxy for urogenital schistosomiasis, Hemastix reagent strips for diagnosis, praziquantel for treatment of cases, and specific forms to report results. After an initial meeting in May 2023, a questionnaire survey and one focus group discussion (FGD) with staff from all 23 health facilities were conducted. All of the 49 health facility staff who were present at the time of the survey were invited to complete a paper-based questionnaire with multiple choice and open-ended questions, including questions about schistosomiasis management in the health facilities (Supplementary file 2). The paper-based data were later entered into an electronic database using ODK.

Once questionnaires had been completed, the FGD was conducted with 10 health facility staff. Participants for the FGD were selected purposively: the local research team had identified staff from 10 health facilities, among which eight were located in hotspots and two in low-prevalence areas, ensuring maximum variation of participants with respect to health facility’s representation, health worker’s role in the health facility, and age and sex. The FGD was conducted in Kiswahili by two members of the research team who were acquainted with social science approaches. The health facility staff, moderator, note taker, and researchers sat on chairs placed around a table with two tape recorders (SONY ICD-PX470, 2021; Beijing, People’s Republic of China). A stopwatch was placed on the centre of the table for the note taker to keep track of the time for the FGD. The moderator facilitated an introduction round and then explained the purpose and procedure of the FGD. Participants gave both written and verbal informed consent for participation, after which the tape recorders were switched on and the stopwatch started. The participants were encouraged to raise their hands if they wanted to speak up and ask questions. The recorded FGD was transcribed using Microsoft Word 2013 (Redmond, WA, USA) and translated from Kiswahili into English.

### Data management

The socio-demographic data, patients’ signs and symptoms, and results of reagent strip testing during the first part of the study were electronically captured using ODK on tablet computers. The urine filtration data were collected on paper and double-entered into a Microsoft Excel spreadsheet. All datasets were cleaned and merged with R version 4.1.3 (www.r-project.org). Each participant was assigned a unique personal identifier code during data collection. Only coded data were used for subsequent analyses.

The quantitative data emanating from health facility staff questionnaires were collected using paper-based questionnaires and later entered into ODK. The data were cleaned with R. The transcript from the FGD was read multiple times and several iterations were made to identify subgroups for participants’ responses.

### Statistical analysis

The statistical analyses of the quantitative data were performed with R. In the first part, all eligible health facility patients who had complete datasets available were included in the parasitological analysis. Participants’ microhaematuria results were graded into five categories: non-haemolysed trace, haemolysed trace, small, moderate, and high. A participant whose urine sample contained at least one *S. haematobium* egg was considered to have an infection. *Schistosoma haematobium* infection was stratified into light-intensity (1–49 eggs/10 ml of urine) and heavy-intensity infection (≥ 50 eggs/10 ml of urine), according to WHO guidelines [34].

The prevalence of microhaematuria and *S. haematobium* in the health facilities was estimated overall and further stratified by area (hotspot and low-prevalence area), sex, and age group (4–5 years: preschool-aged children, 6–12 years: school-aged children, 13–17 years: adolescents, 18–94 years: adults and elderly). Additionally, the results were stratified by educational attainment (current student, no formal education, primary school education, secondary school education, and higher education) and religion (Christians and Muslims). The percentage of patients with self-reported signs and symptoms was calculated and stratified by hotspot and low-prevalence areas and by age group.

In the second part, data for health facility staffs’ knowledge and practices on schistosomiasis management were analysed descriptively and reported as percentages of the number of positive responses amongst all participants who responded to the questionnaire. The transcribed data retrieved from the FGD were coded using Max Weber Qualitative Data Analysis (MAXQDA) Pro (2022, Berlin, Germany) software. A deductive approach was employed to identify subgroups with a common theme [35]. Participants’ quotes were grouped into codes, which captured the main idea for the topics in the FGD. Recurrent codes were further grouped into common themes, which were reported as the research findings, using quoted responses from the health facility staff.

## Results

### Study participation

**Fig. 2:**
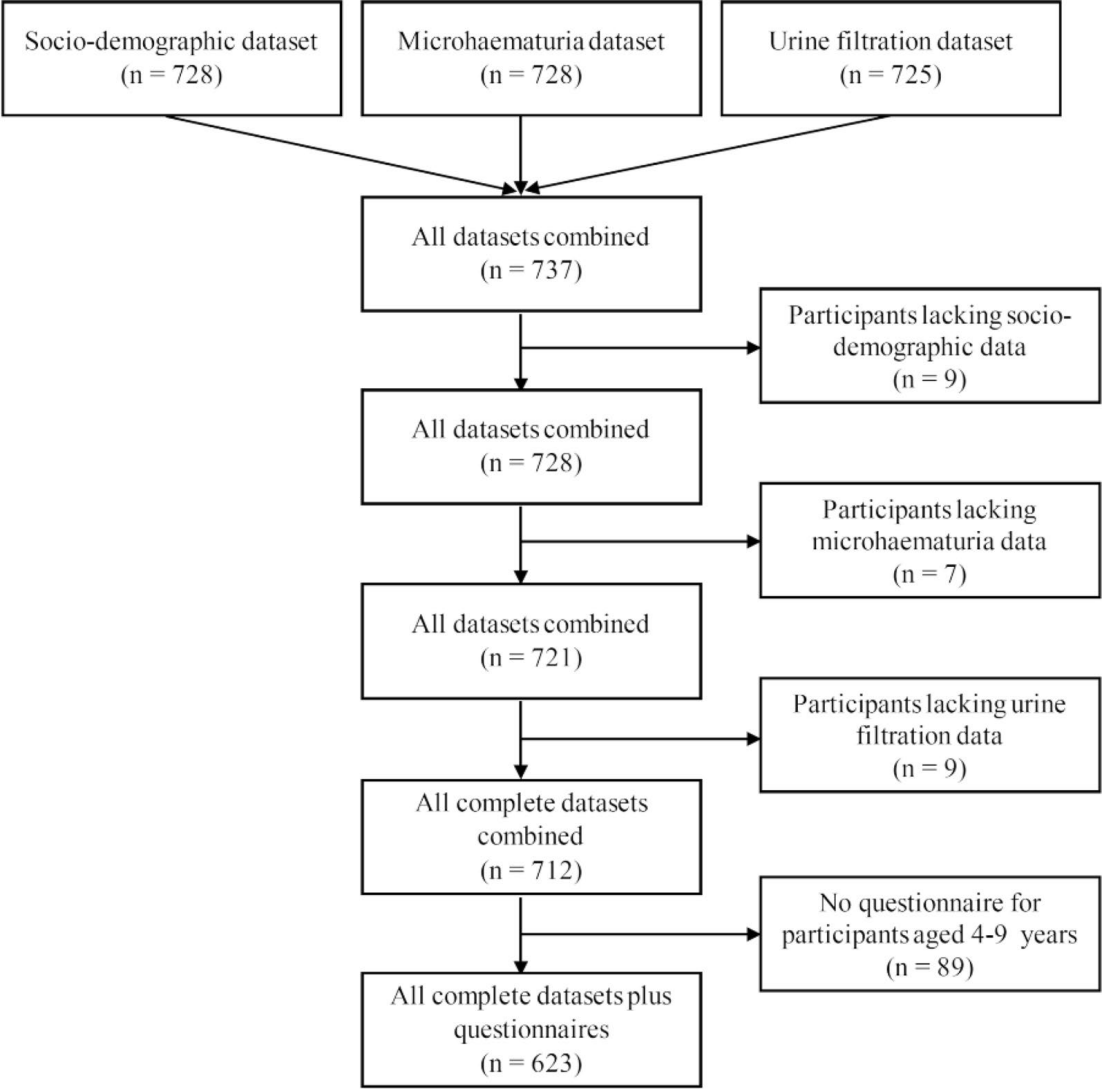
Participation of patients in a health facility-based study conducted in Pemba, Tanzania, in 2023

A total of 737 patients attended one of the four health facilities in Pemba between June and August 2023 and participated in our study (Fig. 2). Twenty-five were excluded for further analysis, as they lacked socio-demographic data (*n* = 9), had no microhaematuria results (*n* = 7), or missed urine filtration results (*n* = 9). The 712 patients with complete data records were subjected to parasitological analysis. Among them, 281 patients attended health facilities in hotspot areas and 431 attended health facilities in low-prevalence areas. Patients aged 4–9 years (*n* = 89) did not respond to the questionnaire about signs and symptoms as per study design. Hence, the analysis of schistosomiasis-related signs and symptoms pertained to the remaining 623 patients aged 10 years and above.

The age of patients ranged from 4 to 94 years with a mean age of 26.2 years. Three-quarter (74.7%) of the patients seeking care were females (532/712), 17.0% had no formal school education (121/712), and 99.4% (708/712) of the patients were Muslims (Table 1).

**Table 1:** Socio-demographic information, microhaematuria and *Schistosoma haematobium* infection status of health facility patients

Characteristics	Total		HF1 (HS)		HF2 (HS)		HF3 (LP)		HF4 (LP)		Microhaematuria		*S. haematobium*	
	*N*	%	*N*	%	*N*	%	*N*	%	*N*	%	*N*	%	*N*	%
Sex														
Female	532	74.7	69	63.9	123	71.1	198	76.4	142	82.6	81	15.2	7	1.3
Male	180	25.3	39	36.1	50	28.9	61	23.6	30	17.4	14	7.8	1	0.5
Age group														
Preschool-age (4–5 years)	31	4.3	7	6.5	6	3.5	12	4.6	6	3.5	1	3.2	0	0.0
School-age (6–14 years)	138	29.4	21	19.4	25	14.4	59	22.8	33	19.2	14	10.1	3	2.2
Adolescence (15–17 years)	64	9.0	12	11.1	14	8.1	23	8.9	15	8.7	6	9.4	1	1.6
Adults and elderly (18–94 years)	479	67.3	68	63.0	128	74.0	165	63.7	118	68.6	74	15.4	4	0.8
Educational attainment														
Students	234	32.9	41	38.0	38	22.0	105	40.5	50	29.1	23	9.8	4	1.8
No formal education	121	17.0	19	17.6	36	20.8	36	13.9	30	17.4	19	15.7	1	0.8
Primary school education	144	20.2	19	17.6	38	22.0	52	20.1	35	20.3	27	18.8	2	1.4
Secondary school education	199	27.9	27	25.0	56	32.4	61	23.6	55	32.0	25	12.6	1	0.5
Higher education	14	2.0	2	1.9	5	2.9	5	1.9	2	1.9	1	7.1	0	0
Religion														
Christian	4	0.4	0	0.0	2	1.2	2	0.8	0	0.0	2	50.0	0	0.0
Muslim	708	99.4	108	100	171	98.8	257	99.2	172	100	93	13.1	8	1.1
Total	712		108		173		259		172		95	13.3	8	1.1

Socio-demographic information, microhaematuria and *S. haematobium* infection status of individuals participating in a health facility-based study conducted in four health facilities in Pemba, Tanzania, in 2023 (HF = health facility, HS = hotspot, LP = low-prevalence)

### Microhaematuria and *S. haematobium* infection

**Fig. 3:**
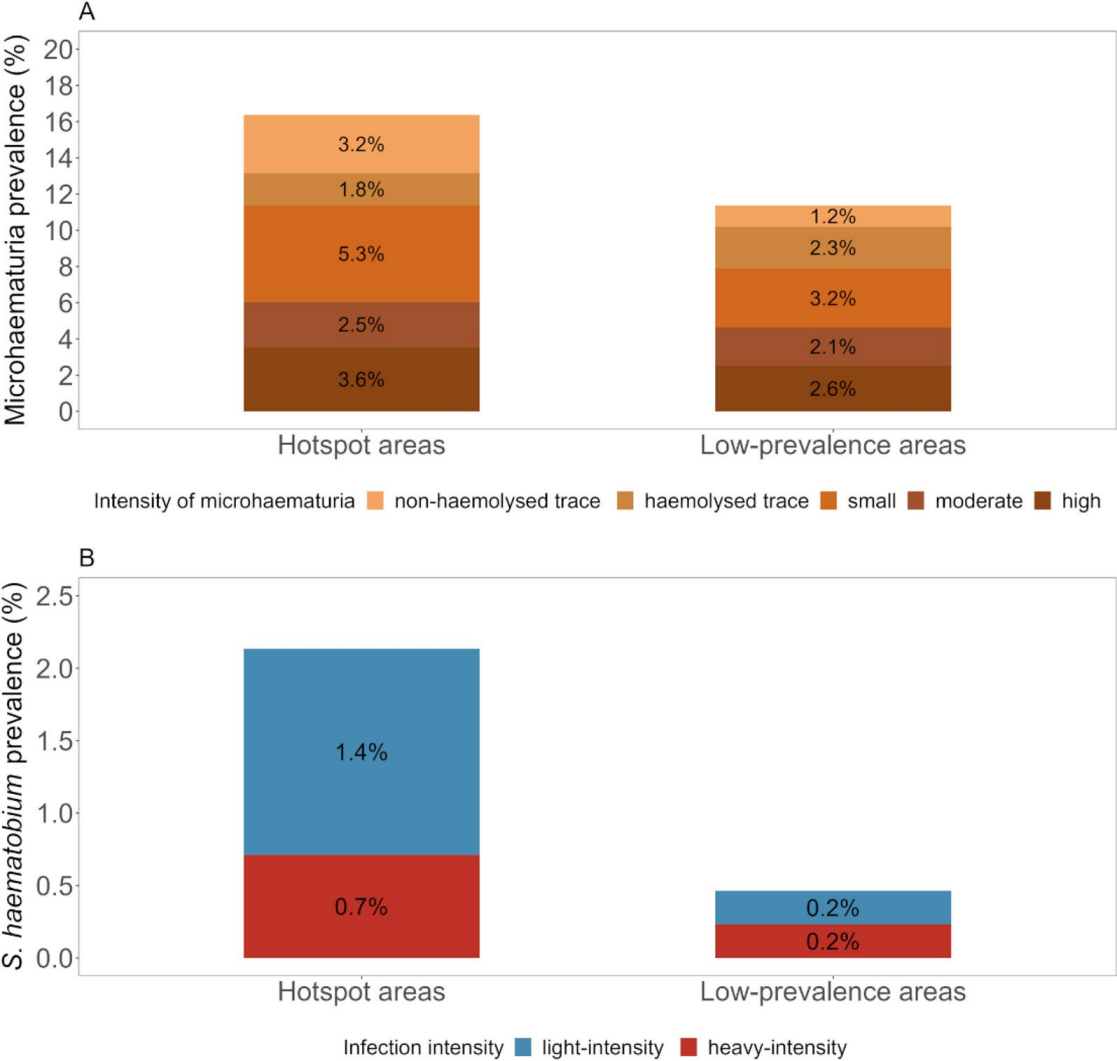
Prevalence of microhaematuria and *Schistosoma haematobium* infections in health facility patients. Microhaematuria (**A**) and *S. haematobium* infections (**B**) in individuals participating in a health facility-based study conducted in Pemba, Tanzania, in 2023, stratified by health facilities located in hotspot (*N* patients: 281) and low-prevalence areas (*N* patients: 431)

Among all health facility patients subjected to parasitological analyses, 13.3% (95/712) were positive for microhaematuria, with grading results ranging from non-haemolysed trace to high (Fig. 3A). The prevalence of microhaematuria in patients seeking care at a health facility in a hotspot area was 16.4% (46/281) and in a low-prevalence area was 11.4% (49/431). A total of 15.2% (81/532) of female patients and 7.8% (14/180) of male participants were microhaematuria-positive. Microhaematuria was present in 3.2% (1/31) of preschool-aged children, 10.1% (14/138) of school-aged children, 9.4% (6/64) of adolescents, and 15.4% (74/479) of adults and elderly.

Eggs of *S. haematobium* were detected in the urine of 1.1% (8/712) of the patients. Among those who tested positive, three had a heavy-intensity infection (≥ 50 eggs/10 ml of urine), while the remaining five had a light-intensity infection (1–49 eggs/10 ml of urine). The *S. haematobium* prevalence in hotspot and low-prevalence health facilities was 2.1% (6/281) and 0.5% (2/431), respectively (Fig. 3B). Female participants were considerably more often *S. haematobium*-positive than male participants [1.3% (7/53) vs. 0.5% (1/180)]. No pre-school-aged children were infected with *S. haematobium*. A total of 2.2% (3/138) of school-aged children, 1.6% (1/64) of adolescents, and 0.8% (4/479) of adults and elderly were infected with *S. haematobium*.

### Schistosomiasis-related signs and symptoms

**Fig. 4:**
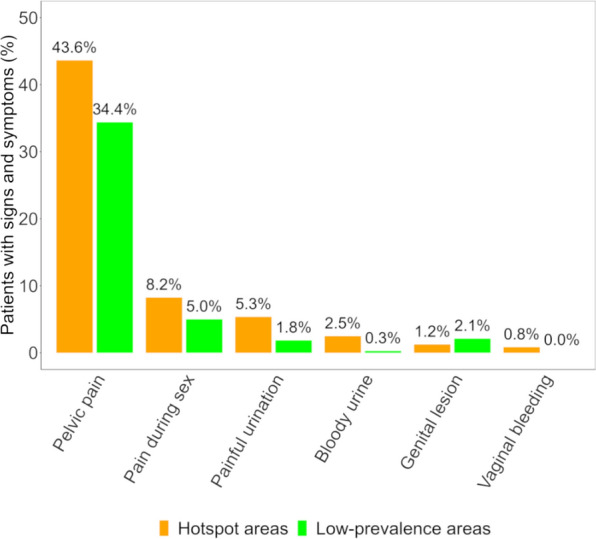
Self-reported signs and symptoms of health facility patients. Signs and symptoms reported by participants of a health facility-based study conducted in Pemba, Tanzania, in 2023, stratified by health facilities located in hotspot and low-prevalence areas

Among the participants with complete demographic and laboratory data, 87.5% (623/712) were interviewed for signs and symptoms of urogenital schistosomiasis. The patients self-reported the following symptoms: 38.0% (237/623) had pelvic pain, 6.3% (39/623) had pain during sex, 3.2% (20/623) had painful urination, 1.8% (11/623) had genital lesions, and 1.1% (7/623) had blood in urine. Stratified by age group, the only symptom reported by school-aged children was pelvic pain (8.0%, 11/80). Among adolescents, pelvic pain was reported by 25.0% (16/64), painful urination by 3.1% (2/64), and genital lesions by 3.1% (2/64). Among adults and elderly, pelvic pain was reported by 43.8% (210/479), pain during sex by 8.1% (39/479), painful urination by 3.8% (18/478%), genital lesions by 1.9% (9/479), blood in urine by 1.5% (7/479), and vaginal bleeding by 0.4% (2/479%). Most of the signs and symptoms were more prominently reported by patients seeking care at a health facility in a hotspot area compared with those reporting at a health facility in a low-prevalence area (Fig. 4).

### Qualifications of health care workers and schistosomiasis-related trainings

Among the health facility staff from the 23 SchistoBreak health facilities who participated in the questionnaire survey, 53.1% (26/49) were males and 46.9% (23/49) were females. The participants were aged between 23 and 60 years (mean age: 32.8 years). In terms of educational attainment, 63.3% (31/49) had a diploma qualification, 26.5% (13/49) worked as diploma nurses, 24.5% (12/49) were consulting doctors, and 20.4% (10/49) were laboratory technicians.

The 10 participants of the FGD were aged between 26 and 45 (mean age: 35.1) years. Seven were females and three were males. The FGD participants consisted of four medical doctors, four laboratory technicians, and two nurses. The FGD participants had all received previous training on the management of schistosomiasis, either within the SchistoBreak project or through other means. The health facility staff said:

“We got our training about how kichocho [Kiswahili term for schistosomiasis] is caused. Also, we get education about the life cycle, treatment of kichocho, complications, if you are treated. How to check kichocho by using Hemastix and how to fill the form” (respondent 7).

“Hmm hmmm, yes there is another thing we get to learn. We get to learn about the situation of kichocho before the project and now how it looks like. Also, they teach us the things the project is doing for the students in school, in the community what they are doing, in different age groups and everyone has to be prevented from kichocho” (respondent 10).

### Knowledge about signs and symptoms for the management of schistosomiasis

In the questionnaire, 89.8% (44/49) of the health facility staff named blood in urine, 87.8% (43/49) painful urination, 81.6% (40/49) pain during sex, and 77.6% (38/49) pelvic/abdominal pain as typical signs and symptoms of urogenital schistosomiasis (Table 2). Most of the health facility staff (40/49, 81.6%) indicated that urine samples are needed to diagnose a patient suspected of schistosomiasis, and 93.8% (46/49) and 59.1% (29/49) named Hemastix reagent strips or microscopy, respectively, as tests for schistosomiasis diagnosis (Table 2).

**Table 2:** Schistosomiasis-related knowledge of 49 health facility staff participating in a questionnaire survey in Pemba, Tanzania in 2023 and needs of health facilities for schistosomiasis management

Variable	Response	Frequency (N)	Percentage (%)
Symptoms of *Schistosoma haematobium* infection	Blood in urine	44	89.8
	Painful urination	43	87.8
	Pain during sex	40	81.6
	Pelvic/abdominal pain	38	77.6
	Problem passing urine	32	65.3
	Genital lesions	29	59.2
	Vaginal bleeding	27	55.1
	Irregular menstruation	21	42.9
Sample collected for diagnosis	Urine	40	81.6
	Urine or stool	3	6.1
	Stool	2	4.1
	Verbal analysis	2	4.1
	Blood	1	2.0
	Blood, urine, stool, and verbal analysis	1	2.0
Method for diagnosis of schistosomiasis	Hemastix reagent strip test	46	93.9
	Urine sedimentation microscopy	29	59.2
	PCR	11	22.4
	Urine filtration	5	10.2
	ELISA	3	6.1
	POC CAA	2	4.1
Treatment against schistosomiasis	Praziquantel	43	87.8
	Praziquantel and albendazole	4	8.2
	No response	2	4.0
HF needs for passive surveillance	Hemastix reagent strip test	40	81.6
	Weekly report forms	35	71.4
	More training	27	55.1
	More laboratory technicians	23	46.9
	Gloves	9	18.4
	More nurses	9	18.4
	Another medical doctor	6	12.2
	Trash cans	6	12.2
	Registers	3	6.1

ELISA, enzyme-linked immunosorbent assay; HF, health facility; PCR, polymerase chain reaction; POC CAA, point-of-care circulating anodic antigen

The FGD confirmed that staff working in the SchistoBreak project health facilities knew about signs and symptoms and diagnosed patients suspected of schistosomiasis using a reagent strip test. Moreover, they expressed awareness of the existence of additional methods for diagnosing urogenital schistosomiasis.

“From my side, when the patient comes, he explains himself by telling the sign he has, for example; when I pee I get pain. Some of them say their pee has blood. So when the patient tells me this, I suspect it can be kichocho but I cannot say, I have to check to confirm. Because in our PHCU we have Hemastix, we asked patients to give us urine and then we check” (respondent 5).

“I understand there is another way to check, using microscope, PCR [polymerase chain reaction] but we do not have” (respondent 3).

### Practices for schistosomiasis management

In the questionnaire, most of the health facility staff (43/49, 87.8%) responded that they administered praziquantel to treat patients with schistosomiasis (Table 2) and that they endeavoured to report the positive cases they diagnose. Participants from the FGD stated as follows:

“Yes, with praziquantel” (respondent 7).

“You measure the height and you know how many medicine you can give and then you ask them to eat while you are watching” (respondent 10).

“We send it on our monthly report and also you come and take the hard copy, and also there is report in the HMIS [health management information system]. Also, we report the case that we suspected in the HMIS, those cases we suspected and that we confirm” (respondent 5).

### Challenges faced by health facility staff in the management of schistosomiasis

The 10 health workers who participated in the FGD reported that they faced different challenges while managing patients with schistosomiasis. Among these challenges were the application of Hemastix reagent strips as a diagnostic tool with insufficient specificity, when microhaematuria is used as a proxy for S. haematobium infection.

“Elder people men, most of time it happens when you are using Hemastix, you can find blood in their urine. But if you make another check with another measurement you will not find kichocho” (respondent 5).

“It depends; it can be the age of the patient. Women can be on her period, men maybe they are overage. It can be his PH [potential of hydrogen] or urethral structure” (respondent 7).

In the FGD, health facility staff also reported that they faced challenges with patient compliance to do a test for schistosomiasis and take the treatment if they are positive. The participants said:

“Whatever, it can be period or another blood in the urine so she feels not comfortable to go pee. Some of them they say do not feel as to do it and that they will come back next time. Or some of them they say they feel pain or some I cannot get urine now. So, they can say like that. But most of them they agree to check, only few of them they refuse” (respondent 3).

“In my PHCU also it is happening like this, they refuse checking and the main reason they said when they find out I am positive I mean they have kichocho, they are afraid to use medicine because they say it is long and it smells bad” (respondent 1).

Some health facility staff mentioned challenges with reporting schistosomiasis cases. One of them said:

“From my side, the challenge I face in my PHCU is when the patient comes and explains the signs and symptoms the other doctors check Hemastix and treat her but they forget to fill the form until I come. And then they inform me that they had one patient that they treat last week but they did not record” (respondent 5).

The health facility staff requested to be informed about the updates on the prevalence of schistosomiasis in the different shehias of the study area to also inform their patients about infection rates in the area.

“When you get the results of kichocho, you have to give us and then we would put on the walls of our PHCU so people will know about the situation of kichocho before and now” (respondent 1).

### Opinions of health facility staff about health facilities’ roles in passive surveillance

Most of the health facility staff who participated in the FGD expressed their interest in actively participating in passive surveillance of urogenital schistosomiasis to support elimination efforts. They reflected that they could play an important role in monitoring and reporting cases via identifying, testing, treating, and carrying out specific education in the health facility and the community while working in collaboration with community heads. Some of them said:

“The PHCU can play a role, for example, when the patient who has the sign first of all they come to the PHCU so when they come here, it is our responsibility when she tells us the symptom you have to check and when you confirm you have to treat the patient so that we can play a big role to make sure that we treat, we give them exact treatment and check our patient” (respondent 10).

“Yes, the health facility can play a role for giving them education in the morning. People can understand and also they can check and get treatment” (respondent 1).

“Please include shehas *[local community leaders]*, at least they can tell people and give education for people to come to the PHCU. Because myself when I am on the road, I see children swimming but they never come here. Very few come, and when they hear that they are going to get medicine, they never show up” (respondent 2).

Health facility staff also stated that they would be able to identify and report outbreaks:

“Yes, they [health facilities] are going to capture the outbreak, but together with the community because as a doctor you only can give them medicine and you can give them exactly as it has to be and you can check everything but if they are still doing the things in the place where they can get kichocho, it is not going to work” (respondent 7).

“For my side, I said we can. Because when they come, in that form, there is information we have to fill for our patient. There is a part asking about the water they are using and they tell us and also tell us the site they are using” (respondent 9).

“I will check people the next month so that I will confirm between this month and the next month and we will know what were the sources” (respondent 1).

### Health facilities’ needs for passive surveillance

Some of the health facility staff who participated in the FGD pointed out that health facilities are not adequately equipped in terms of staffing, diagnostic tools, treatment, and reporting. They expressed a need for improvement in several areas of their health facility. They said:

“If we are using microscope we are going to see the eggs. How many is there, meaning we are going to know much. But if we are using Hemastix, we only know the patient is positive but we do not know like how” (respondent 2).

“I need to add something, when we using microscope, we are going to confirm the diagnosis. It is not like Hemastix that only detects blood in the urine and it is not every blood in the urine that is kichocho, so then if we use microscope we will get confirmation that this is kichocho” (respondent 10).

“We should have enough materials; such as Hemastix, medicine and enough staff” (respondent 10).

“If it is possible, change the design of the medicine” (respondent 6).

Health facility staff also mentioned the need for improvement in health facility infrastructure. They said:

“We do not have laboratory room, we do not have a pharmacy, and we do not have a pharmacist and laboratory technician” (respondent 7).

“Modern tools we have to use, such as a tablet *[computer]*, so that we can send reports to the system” (respondent 3).

## Discussion

This health facility-based mixed-method study confirmed that health facilities can and should play a role in the prevention, diagnosis, management, control, and elimination of schistosomiasis. Indeed, we found that the *S. haematobium* prevalence (1.1%) identified through screening over an 8-day period of all patients attending purposefully selected health facilities was similar to the prevalence (0.8%) reported from recent large-scale community cross-sectional surveys in the same area in Pemba [27]. Hence, determining the *S. haematobium* prevalence via longitudinal passive surveillance or during specific periods or days when people are encouraged to attend health facilities for screening might provide an accurate picture of the endemic situation in a given area.

Our study showed a considerable difference in the microhaematuria (16.4% versus 11.4%) and *S. haematobium* prevalence (2.1% versus 0.5%) of patients attending health facilities located in hotspot or low-prevalence areas. Hence, assessing the *S. haematobium* prevalence in health facilities may provide an opportunity to target interventions according to the local prevalence and, depending on pre-set thresholds, assign either preventive chemotherapy or other interventions tailored to the area. Moreover, by monitoring case numbers in line with the location of occurrence, health facilities could contribute to outbreak detection, followed by programmatic action [18, 20, 21, 36]. By treatment of infected individuals, health facilities automatically contribute to a reduction of morbidity and, as the focus will shift from control to elimination, will assist in breaking transmission.

The patients in our health facility-based study reported different signs and symptoms. Pelvic pain was the most common symptom in both hotspot (37.7%) and low-prevalence (30.4%) health facilities. Painful urination and genital lesions were reported by adolescents and adults but far less frequent (< 5%). Pain during sex, blood in urine, and vaginal bleeding were only reported by adults. In Ghana, a study investigating community health-seeking behaviour suggested that about 20% of community members reported urinary and intestinal-related symptoms to the health facility [17]. These findings imply that health facilities may serve as a reinforcement in the symptomatic identification, testing, and treatment of schistosomiasis, and hence, can contribute to the control and elimination of this NTD [17, 21]. These findings also underpin the WHO recommendation, which suggests that health facilities provide access to treatment with praziquantel to control morbidity due to schistosomiasis in all infected individuals [12].

The health facility staff in the SchistoBreak project study area had good knowledge of the signs and symptoms and the management of urogenital schistosomiasis. These observations are in line with a study conducted in the Mwanza region in the northwestern part of Tanzania in 2020, where most health facility staff reported bloody stool and abdominal pain as typical signs and symptoms of intestinal schistosomiasis caused by *S. mansoni*, the predominant *Schistosoma* species in that area [37]. In Burundi, in contrast, < 10% of the health facility staff were trained in case management of intestinal schistosomiasis, and only a small percentage was aware of the main signs and symptoms of intestinal schistosomiasis [20]. Given these results, it is important to note that the SchistoBreak project provided bi-annual training on the management of urogenital schistosomiasis to health facility staff, which has likely biased our results. In any case, and confirmed also by our FGD, adequate and regular training of health facility staff in the recognition of signs and symptoms and the management of schistosomiasis is warranted, should health facilities play a role in routine treatment of schistosomiasis, as recommended by WHO [12].

The majority of health facility staff in our study knew about, and used, Hemastix reagent strips to detect microhaematuria as a proxy for urogenital schistosomiasis. Positive patients were mostly treated with praziquantel, and treatment was given only after measuring the patient’s height and ensuring that the patient had eaten before drug administration. However, it should be noted that, particularly for the health facilities participating in the SchistoBreak project, there was a constant supply of Hemastix reagent strip tests and praziquantel to the 23 health facilities in the study area for research purposes. In less research-intense settings, reagent strips and praziquantel are not routinely supplied to health facilities and praziquantel tablets might only be managed at the level of NTD control programmes. Yet, our findings concur with previous studies carried out in Burundi and Mali, where healthcare workers were able to identify urogenital and intestinal schistosomiasis cases by using urine reagent strip or the Kato-Katz technique, respectively, and administering praziquantel for positive cases [20, 38]. In a study conducted in the northwestern part of Tanzania, direct smear techniques were most commonly used for diagnosis, and urine reagent strips were only available in 56% (15/27) of researched health facilities [37]. The latter study also reported that only 33.8% of the health facilities had diagnostic services [37]. In Burundi, health facility staff acknowledged praziquantel as the drug of choice for schistosomiasis that is used in preventive chemotherapy campaigns. However, the drug was not available at any level of the health system, even though it was reported to be on the WHO model lists of essential medicines [20, 39]. These findings confirm that, at present, health facilities in many African countries have only a limited capacity to diagnose and manage schistosomiasis [20]. Moreover, it needs to be noted that reagent strips to detect microhaematuria lack specificity and that standard egg microscopy has a limited sensitivity to detect *S. haematobium* infections, particularly in near-elimination settings where infection intensities are mostly low [40]. Hence, the prevalence of microhaematuria and *S. haematobium* infections in our study should be interpreted in light of the limited accuracy of the applied diagnostics. While there are diagnostic approaches that more sensitively detect *S. haematobium* infections, for example, the circulating anodic antigen (CAA) based up-converting phosphor-lateral flow (UCP-LF) assay, they are laboratory tests that require expensive consumables and equipment and are not applicable at the point of care [41–43]. Taken together, there is a pressing need for the development of more sensitive and specific point-of-care diagnostic tools, which can be supplied together with praziquantel to health facilities in Pemba and other African regions if they are supposed to play a role in schistosomiasis control and elimination.

The health facility staff in our study expressed their willingness to participate actively in patient management and passive surveillance of urogenital schistosomiasis and to support the elimination activities in Pemba. They indicated that they would be happy to be assigned an active role in monitoring and reporting cases, test-and-treat activities, outbreak identification, and bolstering health education. They also indicated their readiness to work collaboratively with community leaders. However, they also flagged the challenges in the management of schistosomiasis. These challenges include the limited specificity of microhaematuria assessment for the diagnosis of urogenital schistosomiasis, the limited availability of other diagnostic tests and adequately equipped laboratories for testing, the incompliance of patients with testing and treatment due to the inconvenient size and taste of praziquantel, and an inefficient reporting system.

## Conclusions

Schistosomiasis is a debilitating NTD that is still endemic over large parts of sub-Saharan Africa [1, 3, 6]. Intense control interventions, however, have resulted in a substantial decrease in the prevalence of *Schistosoma* infections in several areas, which are now approaching elimination. In their 2022 guidelines, WHO recommends that health facilities provide access to treatment with praziquantel to control morbidity due to schistosomiasis [12]. Additionally, health facilities should be included as agents for the assignment of targeted interventions, and they can and should play a role in outbreak investigation and passive surveillance for elimination. Our study revealed the willingness of health facility staff and the possibility for primary health care facilities to identify, test, and treat schistosomiasis cases, which would contribute to the control and elimination goals set by WHO. For the effective and sustainable implementation of patient management and passive surveillance into their routine activities, scalable screening pathways need to be identified and the capacities of health facilities need to be further improved by specific staff training and through a supply of accurate point-of-care diagnostic tools and praziquantel for the treatment of cases.

### Supplementary Information

**Supplementary file 1. **Patient questionnaire about signs and symptoms.

**Supplementary file 2. **Health facility staff questionnaire about capacities and needs.

## Data Availability

The datasets used and/or analysed during the current study are available from the corresponding author upon reasonable request.

## Abbreviations

ELISA: Enzyme-linked immunosorbent assay
FGD: Focus group discussion
HF: Health facility
HMIS: Health management information system
ICF: Informed consent form
NTD: Neglected tropical disease
ODK: Open Data Kit
PCR: Polymerase chain reaction
PHCU: Primary health care unit
POC CAA: Point-of-care circulating anodic antigen
WHO: World Health Organization
