# Supplementary material for: Capacities and needs of health care facilities for schistosomiasis diagnosis and management in elimination settings
Source: Parasit Vectors. 2024 Jun 17;17:263. doi: 10.1186/s13071-024-06311-8 (PMC11184784; doi:10.1186/s13071-024-06311-8)
Supplement: Supplementary file 1 — Patient questionnaire about signs and symptoms. [file 13071_2024_6311_MOESM1_ESM.pdf]

Health Facility Patient Questionnaire

District: |__________________| Shehia: |__________________________________| **Participant ID** __ __--__ __ __ __--__ __--__ __ __

Name of health facility: |__________________________________|

Interviewer Name: ____   Date of interview (DD/MM/YYYY): |__|__|/|__|__|/|__|__|__|__|

**Demographics:**		
	1	Name: ____
	2	Sex (M/F): _________
	3	How old are you (in years)? ________
	4	Shehia of residency name: ____
	5	Village of residency name: ____
	6	What religion do you practice? ____Muslim ___Christian ___None ___Other
	7	What is your occupation? ____Health worker ___Police ___Business ___Driver _______Technician ___Housewife ___Ministry ____Farmer ___Fisherman ___Student ___Teacher ____No work ___ Other (specify)____
	8	What is your highest level of education? ______primary: grade 6 _____secondary: form 4 _____secondary: form 6___Bachelor___Master____ Diploma I did not go to school ___ Other (specify)____
**Symptoms related to schistosomiasis:**		
	9	Do you feel pelvic or abdominal pain? ____no ___yes ___I do not know ___I would not like to answer this question
	10	If yes, how long have you had this pelvic or abdominal pain? ____less than 1 week ___ for 1 week ___for 2 weeks ___for 3 weeks _____for 4 weeks ___for more than 1 month ___I do not know___ I would not like to answer the question

	11	Do you have problems passing urine? ____no ___yes ___I do not know ___I would not like to answer this question
	12	If yes, how long have you had problems passing urine? ____less than 1 week ___ for 1 week ___for 2 weeks ___for 3 weeks _____for 4 weeks ___for more than 1 month ___I do not know___ I would not like to answer the question
	13	Do you feel pain during urination? ____no ___yes ___I do not know ___I would not like to answer this question
	14	If yes, how long have you been feeling pain during urination? ____less than 1 week ___ for 1 week ___for 2 weeks ___for 3 weeks _____for 4 weeks ___for more than 1 month ___ I do not know ___I would not like to answer this question
	15	Do you see blood in your urine? ____no ___yes ___ I do not know ___I would not like to answer this question
	16	If yes, how long have you been seeing blood in your urine? ____less than 1 week ___ for 1 week ___for 2 weeks ___for 3 weeks _____for 4 weeks ___for more than 1 month ___ I do not know ___I would not like to answer this question
	17	Do you often have irregular menstruation? ____no ___yes ___I do not know ___I would not like to answer this question
	18	Are you pregnant? ____no ___yes ___ I do not know ___I would not like to answer this question
	19	Are you currently breastfeeding? ____no ___yes ___ I do not know ___I would not like to answer this question
	20	Do you experience vaginal bleeding? ____no ___yes ___I do not know ___I would not like to answer this question
	21	If yes, how long have you been experiencing vaginal bleeding? ____less than 1 week ___ for 1 week ___for 2 weeks ___for 3 weeks _____for 4 weeks ___for more than 1 month ___ I do not know ___I would not like to answer this question
	22	Do you have genital lesion nodules? ____no ___yes ___I do not know ___I would not like to answer this question
	23	If yes, how long have you seen these genital lesion nodules? ____less than 1 week ___ for 1 week ___for 2 weeks ___for 3 weeks _____for 4 weeks ___for more than 1 month ___ I do not know ___I would not like to answer this question
	24	Do you experience pain during sex? ____no ___yes ___I do not know ___I would not like to answer this question

	25	If yes, how long have you been experiencing pain during sex? ____less than 1 week ___ for 1 week ___for 2 weeks ___for 3 weeks ______for 4 weeks ___for more than 1 month ___ I do not know ___I would not like to answer this question
	26	Do you have any other health problem?____no ___yes___ I do not know ___I would not like to answer this question
	27	If yes, which symptom(s)? ____fever___ headache ___vomiting ___nausea _____eye problem ___flue ___ cough ___ Other (specify)___________________________
