# Supplementary material for: Capacities and needs of health care facilities for schistosomiasis diagnosis and management in elimination settings
Source: Parasit Vectors. 2024 Jun 17;17:263. doi: 10.1186/s13071-024-06311-8 (PMC11184784; doi:10.1186/s13071-024-06311-8)
Supplement: Supplementary file 2 — Health facility staff questionnaire about capacities and needs. [file 13071_2024_6311_MOESM2_ESM.pdf]

Swiss TPH

Health Facility Staff Questionnaire

District: |__________________| Shehia: |__________________________________| **Participant ID __ __--__ __ __ __--__ __--__ __ __**

Name of health facility: |__________________________________|

Interviewer Name: ____   Date of interview (DD/MM/YYYY): |__|__|/|__|__|/|__|__|__|__|

**Demographics:**		
	1.	Staff name: ____
	2	How old are you? ____
	3.	Sex (M/F): _________
	4.	What is your function in the health facility? ____community health worker ___diploma nurse ___dentist ___gynecologist _______laboratory technician ___medical doctor ____midwife ___nurse aide ___pharmacist ___surgeon ___ other (specify)____
	5.	What is your highest level of education? ____primary: grade 6 ___secondary: form 4 ___secondary: form 6___bachelor___master____ diploma ___ ___I did not go to school ___ other (specify)____
**Knowledge on schistosomiasis:**		
	6	Have you ever heard about schistosomiasis? ____no ___yes
	7	If yes, where did you hear about schistosomiasis? ____school __radio ___TV ___during training for my profession ____at the health facility staff training and workshop at PHL-IdC___ I do not know ___ other (specify)__________________________
	8	Where do you think schistosomiasis is transmitted? ____field ___toilet __in the river/pond ___trash/dump ____I do not know ___ other (specify)__________________________
	9	During which activities do you think you get schistosomiasis? ____playing with sand ___playing in dirty water __playing in the river or pond ___swimming in the river or pond ___washing with river water ____fishing from river water ___farming with river water __walking barefoot ___I do not know ___ other (specify)__________________________

	10	Do you know which animal is needed for transmission? ____blood fluke ___worm __snail ___bug ___I do not know ___ other (specify)____________________________
	11	Which behavior(s) can help to NOT get infected with schistosomiasis? __not playing in the river/ pond ___not wash in river/pond ____not swim in river/pond ___use tap water/well water __play somewhere else than in the river/pond ___I do not know ___ other (specify)____________________________
	12	Which behavior(s) can help to NOT transmit schistosomiasis? ____not to urinate into river/pond ___take treatment ___I do not know ___ other (specify)____________________________
**Schistosomiasis symptoms:**		
	13	What symptoms of schistosomiasis do you know? ____abdominal pain ___blood in urine ___genital nodule lesion ___irregular menstruation ____pelvic pain ___problems passing urine ___painful urination ___pain during sex ____vaginal bleeding____other (specify) ______________________________________
	14	Do you think pelvic or abdominal pain is a symptom of schistosomiasis? ____no ___yes ____ I do not know
	15	Do you think problem passing urine is a symptom of schistosomiasis? ____no ___yes ____ I do not know
	16	Do you think painful urination is a symptom for schistosomiasis? ____no ___yes ____ I do not know
	17	Do you think blood in urine is a symptom of schistosomiasis? ____no ___yes ____ I do not know
	18	Do you think irregular menstruation is a symptom of schistosomiasis? ____no ___yes ____ I do not know
	19	Do you think vaginal bleeding is a symptom of schistosomiasis? ____no ___yes ____ I do not know
	20	Do you think genital lesion nodule is a symptom of schistosomiasis? ____no ___yes ____ I do not know
	21	Do you think pain during sex is a symptom of schistosomiasis? ____no ___yes ____ I do not know
**Schistosomiasis diagnosis**		

	22	What are the methods used for diagnosing schistosomiasis in your health facility? (multiple answers are allowed) ____ELISA ___haemastix ____ urine sedimentation (microscopy) ____polymerase chain reaction (PCR) ___point of care circulating cathodic antigen (POC-CCA) ____Urine filtration ___I do not know____ Other (specify) ____
	23	Are you aware of any additional diagnostic tests that can be used to test for schistosomiasis? ____no ___yes
	24	If yes, which additional tests are you aware of? ____ELISA ___haemastix ____ microscopy ____polymerase chain reaction (PCR) ___point of care circulating cathodic antigen (POC-CCA) ____urine filtration ___I do not know____ other (specify) ____
	25	Which samples do you collect in your health facility for diagnosis of schistosomiasis ____blood ___stool ____ urine ____verbal analysis only ____ other (specify) ____
	26	Does your health facility have an ultrasound machine? ____no ___yes ____ I do not know
**Treatment**		
	27	Does your health facility staff give treatment to patients diagnosed with schistosomiasis? ____no ___yes ____ I do not know
	28	Which drug does health facility staff give to patients diagnosed with schistosomiasis? ____antibiotics ___albendazole ____herbs ____paracetamol ____praziquantel ___ other (specify) ____
	29	Are patients who receive praziquantel for schistosomiasis treatment in your health facility measured for height? ____no ___yes ____ I do not know
	30	If yes, do health facility staff measure the patient's **height** to give them praziquantel? ____no ___yes ____ I do not know
**Praziquantel Availability**		
	31	Is there praziquantel in stock in your health facility? ____no ___yes ____ I do not know
	32	Has your health facility ever ran out of stock for praziquantel? ____no ___yes ____ I do not know
	33	If Yes, how often does your health facility run out of stock for praziquantel? ____never _______every week ____every month ___less often than once per month ____we never have praziquantel____ I do not know ______other (specify)______________________________

**Referral**		
	34	Does your health facility staff refer patients diagnosed with schistosomiasis? ____no ___yes ____ I do not know
	35	If yes, how often does your staff refer patients diagnosed with schistosomiasis? ____always ___sometimes ____ never ____I do not know
	36	What is the reason for referring patients diagnosed with schistosomiasis? ____because we do not have praziquantel ________other
	37	If other reason, which of these is the other reason for referring patients diagnosed with schistosomiasis? __when the patient is pregnant ____when the patient has other severe illness ___I do not know ___other (please specify)____
	38	To which health facility do you often refer patients diagnosed with schistosomiasis? ____
**Health Facility needs for passive surveillance**		
	39	What in your opinion, is needed for effective management of schistosomiasis at your health facility? ____haemastix tests___praziquantel ____weekly report forms ____trash can ___gloves ____more training ___registers ____more nurses ____more laboratory technicians ___Another medical doctor? ____other (specify) ____
